# Patellar Tendon Reconstruction Using Peroneus Longus Tendon Autograft Following Revision Knee Arthroplasty: A Case Report

**DOI:** 10.7759/cureus.41052

**Published:** 2023-06-27

**Authors:** Ahmet Serhat Genc, Nizamettin Güzel, Korkut Arar, Anil Agar

**Affiliations:** 1 Orthopaedics and Traumatology, Samsun Training and Research Hospital, Samsun, TUR; 2 Orthopaedics and Traumatology, Samsun Gazi State Hospital, Samsun, TUR; 3 Orthopaedics and Traumatology, Firat University, Faculty of Medicine, Elazığ, TUR

**Keywords:** knee, autograft, revision arthroplasty, peronous longus, patellar tendon rupture

## Abstract

Patellar tendon rupture is a rare but serious complication resulting in loss of knee extension that may develop during and after total knee arthroplasty and negatively affects the patient's quality of life. There are a number of surgical options available, from primary repair to reconstruction. Peroneus longus tendon autograft has begun to be used for knee extensor mechanism repair in recent years. In this case report, we aimed to present the case of a patient with traumatic patellar tendon rupture after revision knee arthroplasty. In conclusion, patellar tendon reconstruction using peroneus longus tendon autograft can be considered a successful method in selected patients following knee arthroplasty. It allows early rehabilitation via stable graft fixation and provides good clinical and functional outcomes in the late period.

## Introduction

Patellar tendon rupture is one of the most common causes of extensor dysfunction with patellar fracture and quadriceps tendon rupture. Although patellar tendon rupture after total knee arthroplasty (TKA) is a rare complication (0.17%), it is a serious complication that causes poor prognosis, and it can cause a serious decrease in performance and quality of life in patients [1,2]. Traumatic or atraumatic causes can be listed in their etiology. Atraumatic ruptures may often occur in cases that affect tendon quality, such as systemic inflammatory and endocrine diseases, diabetes mellitus, chronic renal failure, and steroid use [3-6]. Traumatic ruptures usually develop with a blow to the knee, commonly resulting from eccentric loading [7]. Multiple surgical interventions may damage the vascular anastomosis around the knee, resulting in decreased and weakened blood flow to the surrounding tissue [8], and the knee is also at risk due to multiple surgeries and increased tissue scarring [9,10]. Patellar tendon rupture may occur due to surgical technique-related failures such as component malposition [1].

Patellar tendon ruptures that occur within three months after TKA are considered early injuries, while all other injuries are considered late injuries [11]. Delayed and neglected patellar tendon ruptures require performing different surgical procedures. Non-surgical treatment of these injuries usually results in chronic pain, walking difficulties, extensor delay, decreased range of motion, limb instability, and increased risk of falling [12]. Patellar tendon ruptures usually require graft reconstructions (synthetic, allograft, and autograft), with or without augmentation, and primary repair with augmentation. However, there is no consensus on which provides the best clinical outcomes [2,13]. The use of peroneus longus tendon autograft has not been reported yet for patellar tendon reconstruction. In this case report, our aim was to present the patient who underwent patellar tendon reconstruction using peroneal tendon autograft following revision knee arthroplasty; owing to this method, we achieved excellent functional results during a six-month follow-up period.

## Case presentation

**Figure 1:**
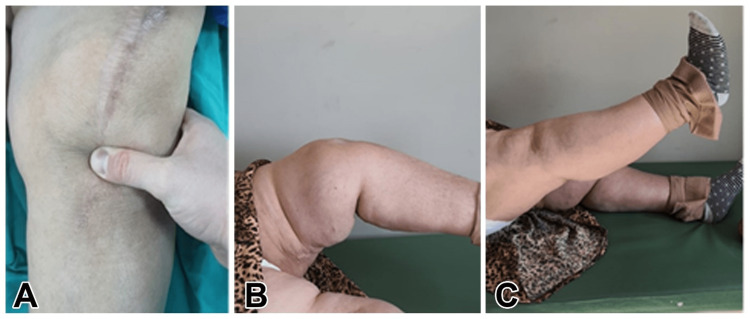
A: Preoperative patellar tendon gap image. B: Insufficiency of extension on the ruptured side of the preoperative patellar tendon. C: Full-extension view of the healthy knee

A 74-year-old woman with aseptic loosening of total knee replacement in his left knee had a revision TKA (Tıpsan Medical Devices Company, Turkey) of her left knee using the medial parapatellar approach in July 2022. Postoperatively, the range of motion of the knee was from 0 degrees to 120 degrees. The patient fell down, hurting her operated knee two months after the operation. After this injury, she was unable to extend her knee, and a radiograph showed a superior migration of the patella compared to the contralateral knee. Further, there was a 2 cm gap in the patellar tendon under palpation (Figure 1). 

**Figure 2:**
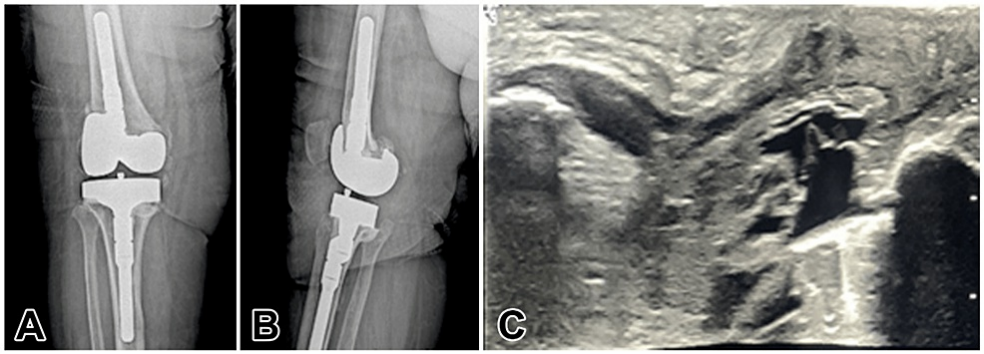
A: Anteroposterior knee radiograph of the side with preoperative patellar tendon rupture. B: Lateral knee radiograph of the side with preoperative patellar tendon rupture. C: Patellar tendon rupture USG image USG, ultrasonography.

In the radiological examination, according to the Insall-Salvati method, the length of patella/length of the patellar tendon (LT/LP) ratio was 1.8 in the lateral radiograph of the left knee and [1,2] in the right knee, which was normal (Figures 1, 2). Patellar tendon continuity could not be observed completely in the superficial ultrasonography (USG).

Informed consent was obtained from our patient for the use of clinical details, imaging data, and clinical photographs, and this consent was obtained before this case report was written and documented in our electronic medical records.

Surgery

A medial parapatellar arthrotomy was performed through the old incision. When the patellar tendon was reached, mid-substance a total tear of the patellar tendon was seen at the knee joint level, and the space between was found to be filled with scar tissue. The ruptured patellar tendon ends debrided, the scar tissue between these was cleaned, and it was seen that the patella could easily be lowered down. The peroneus longus tendon was then harvested from the ipsilateral extremity with a length of approximately 27 cm. The tendon was cleaned of remaining muscle and fatty tissue and both the free ends of the tendon were securely fixed to each other with a Bunnell’s stitch of nonabsorbable suture material.

**Figure 3:**
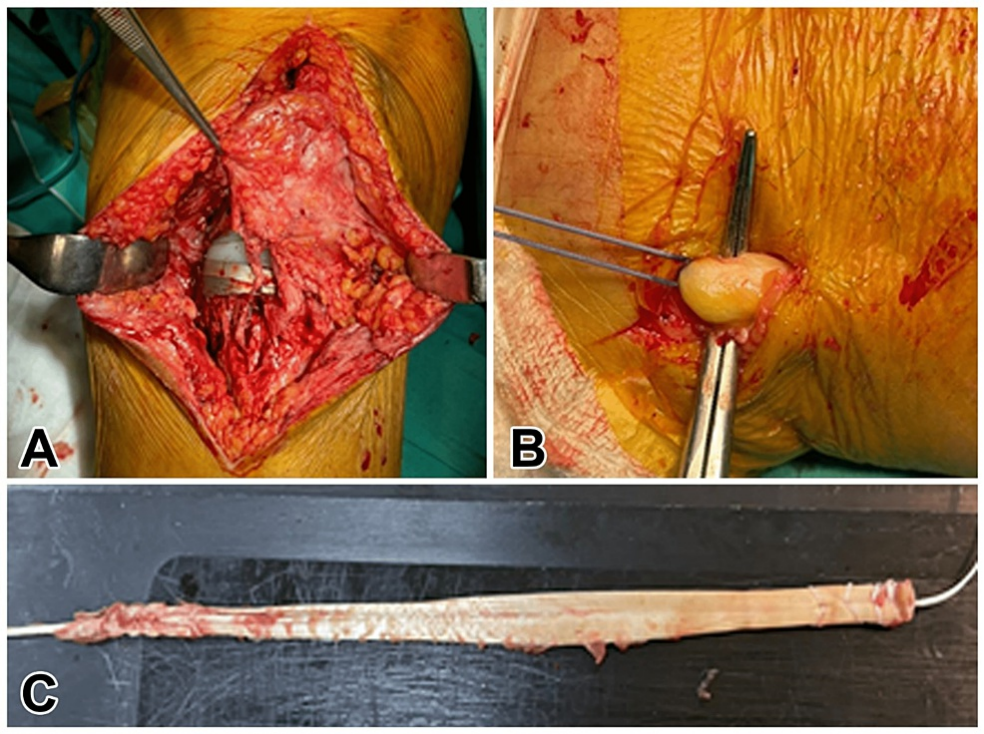
A: Appearance of patellar tendon rupture after arthrotomy. B: The moment of removal of the peroneus longus tendon. C: Preparation of the peroneus longus tendon

**Figure 4:**
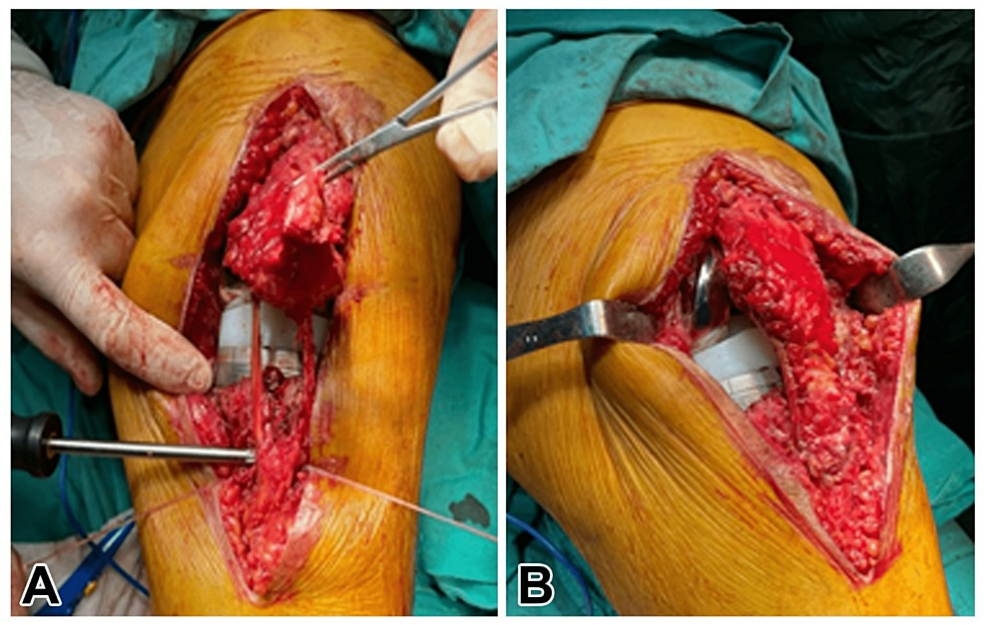
A: Image of the peroneus longus tendon during repair. B: Fixation of the peroneus longus tendon to the patellar tendon after repair

**Figure 5:**
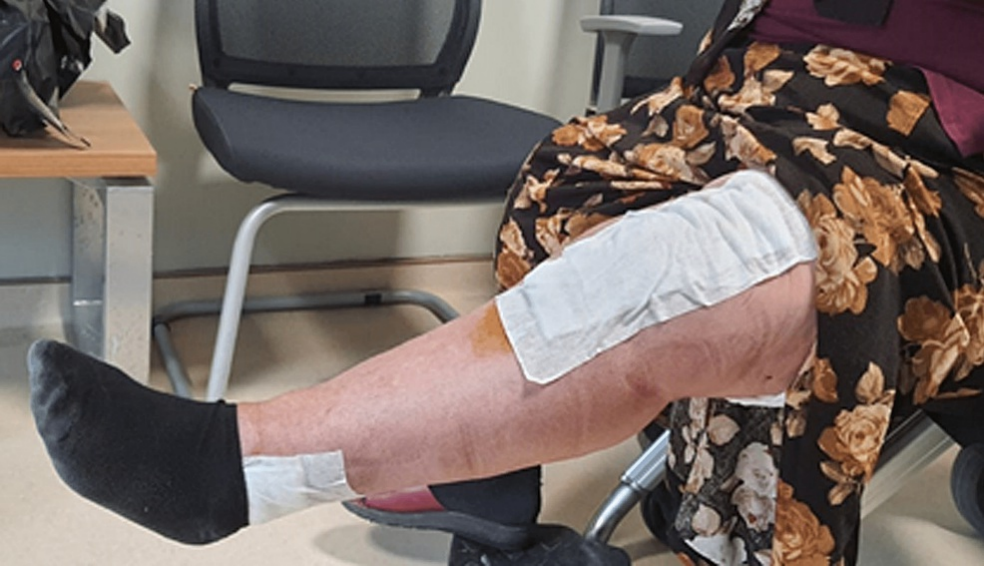
The amount of active extension when the patient's sutures were removed 15 days postoperatively

Next, a drill hole was made in the tuberositas tibia. The drill hole was made just large enough to allow the tendons to pass (6 mm in width). The peroneal tendon was removed by passing one end from the canal in the tibial tuberosity from lateral to medial, and from the canal in the patella from medial to lateral, and the same tendon end was passed from lateral to medial through the tunnel in the tuberosity (Figure 3). Then the final tensioning of the graft was performed ensuring the correct height of the patellar position at 30 degrees of flexion and one bioscrew was fixed into the tibial tunnel (Figure 4). The graft was fixed with a 6 mm PEEK (poly-ether-ether-ketone) polymer interference screw used routinely in the fixation of the anterior cruciate ligament reconstruction. Following this, the remaining parts of the patellar tendon were sutured to the peroneal tendon with an ethibond suture. The layers were closed as appropriate after observing joint movements (Figure 5).

Clinical follow-up

The patient was kept in a long leg splint in full extension for 15 days following the operation. On day 15, the leg was placed in a hinged knee brace, and active quadriceps exercises between 0° and 10° were started. The amount of flexion was increased by 10° per week. Active knee range of motion was 0-90° at week 10. The crutches were used for balance with weight bearing as tolerated for the first eight weeks. Active knee movements were between 0° and 130° at the six-month follow-up, and the patient’s gait was normal and painless. There was 3 cm atrophy in the quadriceps; however, the muscle strength was at 5, and the preoperative clinical findings had disappeared. In the 12-month control, the active range of motion of the knee was between 0° and 130°. There was a 1.3 cm permanent quadriceps atrophy. No patellar tendon re-rupture or postoperative infection occurred. The patient had good quadriceps muscle strength and she could climb the stairs unaided. The patient was satisfied with the functional result. The average Knee Society Score (KSS) for function improved from 14 points preoperatively to 90 points at the final follow-up.

## Discussion

Numerous surgical techniques have been described for patellar tendon rupture in TKA; however, few have safely restored a functional extensor mechanism [1,2]. In knees that have not undergone arthroplasty, primary repair with fixation with sutures, staples, and isolated suture fixation has been the first treatment option in patellar tendon rupture, and encouraging results have been reported [14]. Primary repair attempts after TKA have rarely rendered the extensor function [9,10]. Primary repair is the best option in cases of acute rupture with sufficient tendinous tissue to allow for repair. A study evaluating 18 knees treated for patella tendon rupture after TKA reported that direct repair of extensor mechanism disruption was associated with inconsistent outcomes, with only 25% of patients having a successful outcome, and staple fixation giving the best results [15]. Therefore, the use of suture fixation alone is not safe.

In patients who experience impaired extensor mechanisms after TKA, graft reconstruction can restore the knee joint effectively and reliably and improve functional status [16]. Different types of autogenous tissue grafts that can be used to strengthen extensor mechanism reconstruction include semitendinosus tendon, gracilis tendon, quadriceps tendon turndown flap, free fascia lata graft, and gastrocnemius rotational flap [17,18]. Spoliti et al. [16] reported an excellent result in nine patients who used autologous ipsilateral hamstring tendon grafts and found that the graft was more robust than other autograft types. However, these autografts may not be found in patients who have had more than one knee surgery. Hamstring autograft tendons may also be of unreliable length, thickness, strength, and integrity. In peroneal tendon autograft, reconstruction can be achieved with a solid tendon of desired length and thickness.

Despite potential drawbacks, allograft repair is currently the most consistent surgical technique. In a systematic review by Maffulli et al. [19], the results of 254 patients who underwent allograft repair were reviewed and the results showed good clinical outcomes with careful surgical technique. Autografts are used to eliminate the risk of contagious infection and allograft autoimmune reactions that can occur in allograft reconstructions.

Barrack et al. [13] successfully used Achilles tendon allografts with calcaneal bone block or extensor mechanism allografts consisting of quadriceps tendon-patella-patella tendon-tibial-tubercle. If the quadriceps mechanism was chronically impaired then the patella was drawn to extreme proximal in patellar tendon rupture that developed in TKA [16,20]. Achilles tendon allograft is preferred because it is less voluminous and provides the advantage of longer length for safer graft anchorage, especially in chronic extensor mechanism impairment where tendon retraction is important [20].

The use of synthetic meshes over conventional allografts has increased in recent years due to commercial availability, absence of donor site morbidity, and reduced risk of host immune reactions and disease transmission [21]. They allow for a significant part of the load to be carried by the synthetic ligament in the early postoperative period and provide a gradual transfer of the load to the repaired tissue while the primary tissue heals. Concerns with this implant include an increased risk of infection and poor tissue retention in patients who have had prior TKA revisions [22].

The goal of treatment in patellar tendon reconstruction is the functional and structural restoration of the extensor mechanism in order to achieve an active knee extension. In this case report, we reported a patient with patellar tendon rupture, in whom we performed patellar tendon reconstruction with peroneus longus tendon autografts. With a one-step surgery, we achieved functional restoration using a strong and long tendon autograft. The peroneus longus tendon was of the required length and strength compared to the hamstring tendons. As it was an autograft, we have excluded the potential issues which might occur with allografts, including infection, rejection, as well as high costs of treatment.

## Conclusions

In conclusion, patellar tendon reconstruction using peroneus longus tendon autograft can be considered a successful method in selected patients following knee arthroplasty. It allows early rehabilitation via stable graft fixation and provides good clinical and functional outcomes and less infection risk in the late period. Future studies should be evaluated with more patients and follow-up periods, and different autograft methods should be evaluated.
